# Ameliorative Effects of Dimetylthiourea and N-Acetylcysteine on Nanoparticles Induced Cyto-Genotoxicity in Human Lung Cancer Cells-A549

**DOI:** 10.1371/journal.pone.0025767

**Published:** 2011-09-29

**Authors:** Ritesh Kumar Srivastava, Qamar Rahman, Mahendra Pratap Kashyap, Mohtashim Lohani, Aditya Bhushan Pant

**Affiliations:** 1 Indian Institute of Toxicology Research, Lucknow, India; 2 Council of Scientific and Industrial Research, New Delhi, India; 3 Department of Biotechnology, Integral University, Lucknow, India; Johns Hopkins School of Medicine, United States of America

## Abstract

We study the ameliorative potential of dimetylthiourea (DMTU), an OH^•^ radical trapper and N-acetylcysteine (NAC), a glutathione precursor/H_2_O_2_ scavenger against titanium dioxide nanoparticles (TiO_2_-NPs) and multi-walled carbon nanotubes (MWCNTs) induced cyto-genotoxicity in cultured human lung cancer cells-A549. Cytogenotoxicity was induced by exposing the cells to selected concentrations (10 and 50 µg/ml) of either of TiO_2_-NPs or MWCNTs for 24 h. Anti-cytogenotoxicity effects of DMTU and NAC were studied in two groups, i.e., treatment of 30 minutes prior to toxic insult (short term exposure), while the other group received DMTU and NAC treatment during nanoparticles exposure, i.e., 24 h (long term exposure). Investigations were carried out for cell viability, generation of reactive oxygen species (ROS), micronuclei (MN), and expression of markers of oxidative stress (HSP27, CYP2E1), genotoxicity (P^53^) and CYP2E1 dependent n- nitrosodimethylamine-demethylase (NDMA-d) activity. In general, the treatment of both DMTU and NAC was found to be effective significantly against TiO_2_-NPs and MWCNTs induced cytogenotoxicity in A549 cells. Long-term treatment of DMTU and NAC during toxic insults has shown better prevention than short-term pretreatment. Although, cells responded significantly to both DMTU and NAC, but responses were chemical specific. In part, TiO_2-_NPs induced toxic responses were mediated through OH^•^ radicals generation and reduction in the antioxidant defense system. While in the case of MWCNTs, adverse effects were primarily due to altering/hampering the enzymatic antioxidant system. Data indicate the applicability of human lung cancer cells-A549 as a pre-screening tool to identify the target specific prophylactic and therapeutic potential of drugs candidate molecules against nanoparticles induced cellular damages.

## Introduction

Intracellular biological interactions of metal oxide nanoparticles and carbon nanotubes have been shown in the literature and marked alarming to the safety [1], [2]. Titanium dioxide nanoparticles (TiO_2_ -NPs) and multiwalled carbon nanotubes (MWCNTs) are known to induce cytotoxic and genotoxic responses in both *in vitro* [3], [4], [5] and i*n vivo* models [6], [7]. In general, ROS generation mediated oxidative stress and impaired antioxidant status has been suggested as major cause of cytotoxicity and genotoxicity of nanoparticles [8], [9], [10]. Besides, the number of factors including particle size, chemical composition and surface charges etc. have also play role to determine the extent of nanoparticles toxicity [11], [12], [13], [14]. Nanoparticles induce ROS generation by the activation of enzymes of cytochrome P450s family [15], [16] and/ or by disrupting mitochondrial electron transport chain function [9]. The association of ROS generation with induced levels of heat shock protein 27 (HSP27), cytochrome P450 2E1 (CYP2E1) and P^53^ have been established as markers of chemical induced oxidative stress, apoptosis and genotoxicity [10], [17], [18].

One of the effective ways to prevent the ROS mediated cellular injury is dietary or pharmaceuticals augmentation of free radical scavengers. Hence, attempts have been made to prevent/ restore the nanoparticles induce cytogenotoxicity using radical scavengers, such as desferoxamine [8], antioxidants - resveratrol [19] and N-acetlylcysteine (NAC) [4], [20], [21], in variety of cell systems of human and animal origin. However, changes in the levels of expression (mRNA and protein) of markers involved in oxidative stress and genotoxicity in cultured cells exposed to nanoparticles have not been studied so far. Thus, the present investigations were aimed to study the mechanistic insights of TiO_2_-NPs and MWCNTs induced oxidative stress and cyto-genotoxicity in human lung cancer cells A549 and ameliorative effect of free radical trapper and antioxidants DMTU and NAC respectively. DMTU, was selected in the study, since this thiogroup containing compound has great reactivity toward OH^•^ radicals due to the extremely favorable electron-donating characteristics of the sulfhydryl group; thus, known as potential hydroxyl radical scavenger [22]. The other compound used i.e., NAC is a precursor of glutathione (GSH), is an important cellular antioxidant and known to play a key role in the protection of cells against oxidative stress by inhibiting the formation of H_2_O_2_ under in vitro conditions [23]. NAC is also known to improve cellular GSH level by directing cystine into the GSH synthesis pathway, because cystine is a common precursor of both NAC and GSH [24].

## Results

### Characterization of nanoparticles

**Figure 1:**
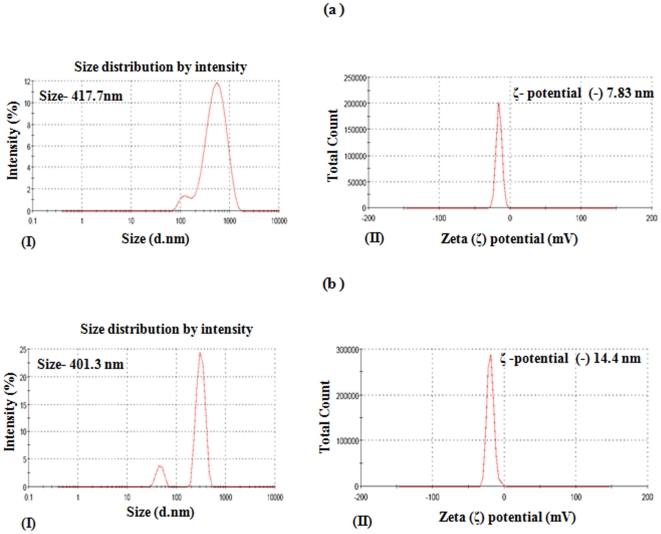
Characterization of TiO_2_-NPs and MWCNTs using Dynamic Light Scattering. Size distribution and zeta potential of TiO_2_-NPs (a, I & II) and MWCNTs (b, I & II) were determined using dynamic light scattering and phase analysis light scattering (PALS) in a Zetasizer Nano-ZS, Model ZEN3600.

**Figure 2:**
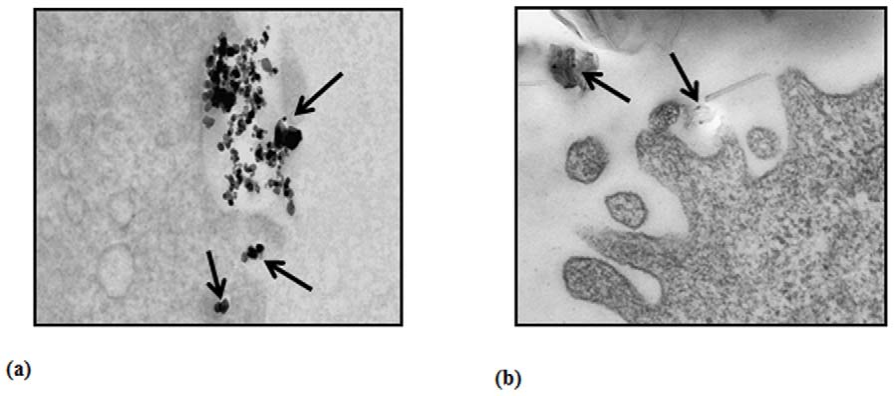
Transmission Electron Microscopy (TEM) analysis of internalization of TiO_2_-NPs in A549 cells. TEM microphotographs showing internalization of TiO_2_-NPs (a, b) in human lung cancer cells-A549. Arrows indicates the nanoparticles were adhered on the cell surface between microvilli and pseudopodes within few minutes of time (a) and subsequently internalized in small vacuoles deep in the cytoplasm, when observed at 24 h (b).

The mean hydrodynamic diameters of the TiO_2_-NPs and MWCNTs suspension in complete medium were 417.7 nm and 401.3 nm, and zeta (ζ) potentials were calculated as (-) 7.83 mV and (-) 14.4 mV respectively (Figure 1 a (I,II) & b (I,II)). The TEM studies show the particle size range from 5-20 nm in diameter and 300–2000 nm in length for MWCNTs (data have already been published by us) [15]. TiO_2_-NPs (anatase) used in the study were of 99.7% purity and the particle size ranges between 5–25 nm in TEM studies. The particles were adhered on the cell surface between microvilli and pseudopodes, when incubated for 24 h, subsequently internalized in small vacuoles at cortical cytoplasm (Figure 2 a & b). We didn't observe any significant change in hydrodynamic particle (TiO_2_-NPs and MWCNTs) size in the presence of DMTU and NAC during DLS measurement. After sterilization, endotoxin was not detected in both the nanoparticles by LAL test. The detection limit was 0.03 EU/ml.

### Restoration of cell viability

**Figure 3:**
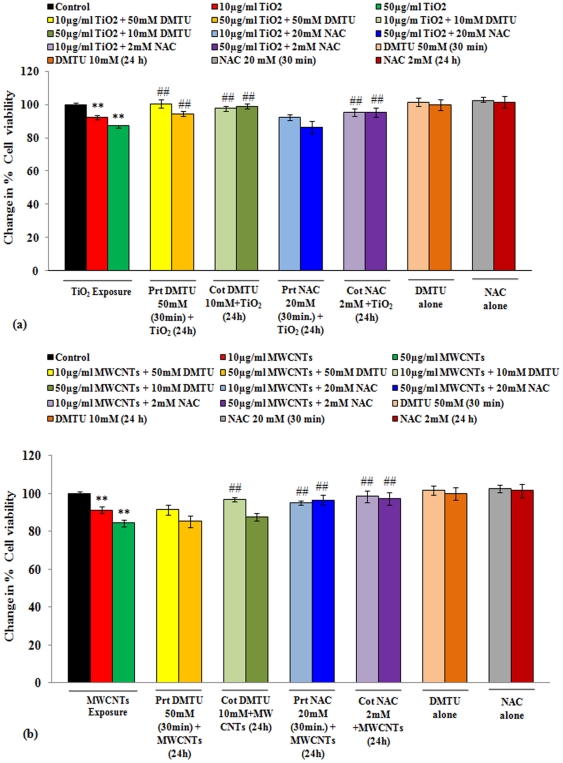
Cell viability restoration by DMTU and NAC in human lung cancer cells-A549 receiving the exposure of TiO_2_-NPs or MWCNTs. (**a**) In short term group, cells were pre-treated with either of DMTU or NAC for 30 minutes followed by exposure of TiO_2_-NPs (10 & 50 µg/ml) for 24 h. In long term group, cells were receiving a co-exposure (24 h) of DMTU/NAC and TiO_2_-NPs (10 & 50 µg/ml). Cells were then analyzed for DMTU/NAC mediated restoration in the cell viability reduced due to TiO_2_-NPs exposure. All values are mean±S.E. of 3 experiments. ^**^P<0.01 (unexposed control Vs TiO_2_-NPs exposure). ##P<0.01 (TiO_2_-NPs exposure Vs DMTU/NAC treatment). (**b**) In short term group, cells were pre-treated with either of DMTU or NAC for 30 minutes followed by exposure of MWCNTs (10 & 50 µg/ml) for 24 h. In long term group, cells were receiving a co-exposure (24 h) of DMTU/NAC and MWCNTs (10 & 50 µg/ml). Cells were then analyzed for DMTU/NAC mediated restoration in the cell viability reduced due to MWCNTs exposure. All values are mean ± S.E. of 3 experiments. ^**^P<0.01 (unexposed control Vs MWCNTs exposure). ##P<0.01 (MWCNTs exposure Vs DMTU/NAC treatment).

The trend for restoration in cell viability was similar for all the three assays used in the study. However, MTT assay was found to be most sensitive when compared with the other assays viz., NRU and released LDH. The results of DMTU or NAC induced restoration in the percent viability of A549 cells exposed to nanoparticles are summarized in Figure 3 a & b. The short term pretreatment (30 minutes) of DMTU shows significant protection in viability of A-549 cells exposed to TiO_2_-NPs (10, 50 µg/ml), whereas the response of NAC pretreatment was non-significant against TiO_2_-NP induced loss of cell viability. Cells receiving long term (24 h) exposure of DMTU (10 mM) or NAC (2 mM) along with TiO_2_-NPs (10, 50 µg/ml) exposure have shown more or less similar magnitude and trends as observed in short term exposure (Figure 3 a). Contemporary to this, short term exposure of NAC was found to be significantly effective against MWCNTs exposure, whereas, DMTU was not effective. Long term exposure of both NAC and DMTU has shown similar trend and efficacy against MWCNTs exposure, as was in case of short term exposure (Figure 3 b). In general, long term exposure of DMTU and NAC has shown little better restoration in cell viability than short term.

### ROS generation

**Figure 4:**
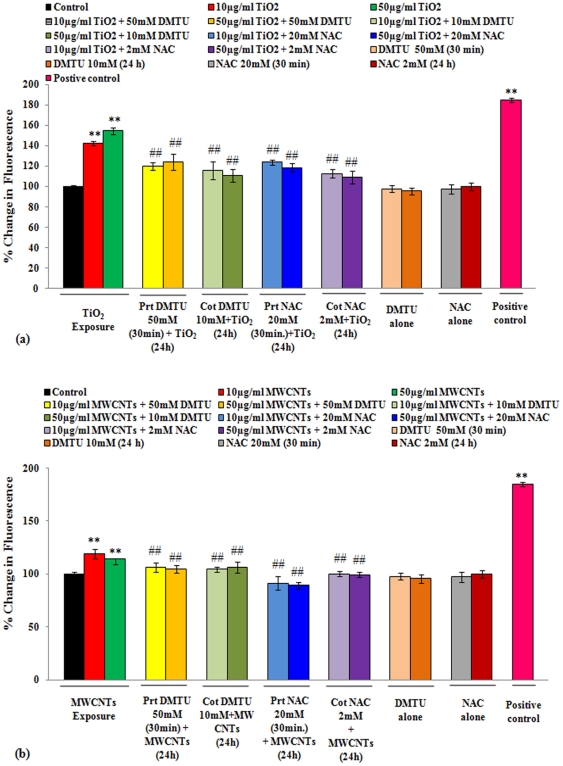
Effect of DMTU/NAC on the restoration of ROS generated in A549 cells exposed to TiO_2_-NPs/ MWCNTs. ROS generation was detected using ‘2, 7-dichlorofluorescin diacetate (DCFH-DA) dye. (**a**) In short term group, cells were pre-treated with either of DMTU or NAC for 30 minutes followed by exposure of TiO_2_-NPs (10 & 50 µg/ml) for 24 h. In long term group, cells were receiving a co-exposure (24 h) of DMTU/NAC and TiO_2_-NPs (10 & 50 µg/ml). Cells were then analyzed for DMTU/NAC mediated restoration in the levels of intracellular ROS, which was induced by the exposure of TiO_2_-NPs. All values are mean ± S.E. of 3 experiments. ^**^P<0.01 (unexposed control Vs TiO_2_-NPs exposure). ##P<0.01 (TiO_2_-NPs exposure Vs DMTU/NAC treatment). The positive control group was consisting of A549 cells pre-treated (1h) with 500 µM of H_2_O_2_. (**b**) In short term group, cells were pre-treated with either of DMTU or NAC for 30 minutes followed by exposure of MWCNTs (10 & 50 µg/ml) for 24 h. In long term group, cells were receiving a co-exposure (24 h) of DMTU/NAC and MWCNTs (10 & 50 µg/ml). Cells were then analyzed for DMTU/NAC mediated restoration in the levels of intracellular ROS, which was induced by the exposure of MWCNTs. All values are mean ± S.E. of 3 experiments. ^**^P<0.01 (unexposed control Vs MWCNTs exposure). ##P<0.01 (MWCNTs exposure Vs DMTU/NAC treatment). The positive control group was consisting of A549 cells pre-treated (1h) with 500 µM of H_2_O_2_.

The statistically significant (p<0.01) ROS generation was observed in A549 cells receiving TiO_2_-NPs and MWCNTs (10 & 50 µg/ml) for 24 h. Both short term (30 min) and long term (24 h) treatment of DMTU and NAC was found to be effective significantly (p<0.01) in diminishing the ROS levels. However, the magnitude of reduction of ROS levels was greater in long term treatment. Long term treatment of NAC was more effective than DMTU (Figure 4 a & b). We did not observe any increase in fluorescence intensity due to suspended nanoparticles in the culture medium (without cells). Similarly, no fluorescence increase was observed with DCFH-DA dye alone in the culture medium (without cells). The observations indicate that the alterations in the fluorescence intensity were because of intracellular ROS generated in A549 cells only.

### Cytokinesis blocked micronucleus assay (CBMN-assay)

**Figure 5:**
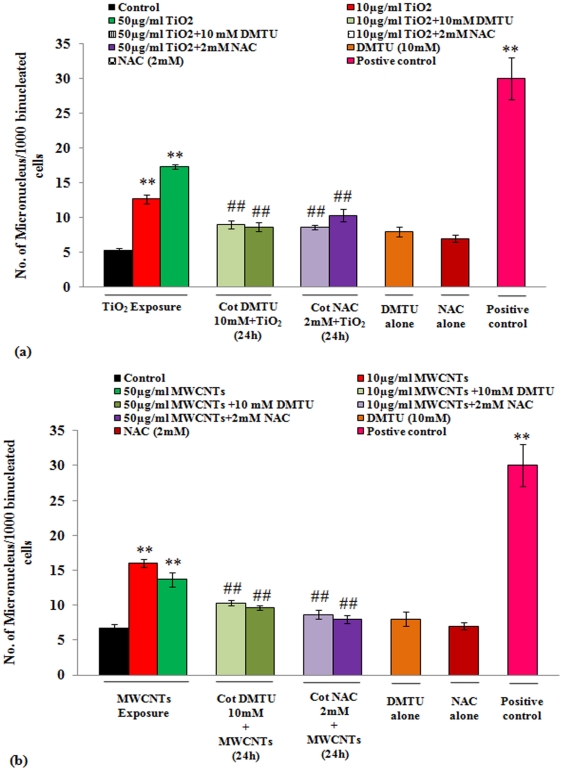
Effect of DMTU/NAC on the reduction of micronuclei formation in A549 cells exposed to TiO_2_-NPs/ MWCNTs. (**a**) The cells were exposed to TiO_2_-NPs (10 & 50 µg/ml) with or without DMTU (10 mM) and NAC (2 mM) for 24 h. Ethyl methanesulfonate (6mM) was used as a positive control. Each data point represents the mean of three slides. A total 1000 bi-nucleated (BN) cells with well-defined cytoplasm were scored. Parallel sets were also run under identical conditions by exposing the cells only with either of DMTU or NAC. This group was used to compare the data between scavenger treated Vs non-scavenger treated cells. All values are mean ± S.E. of 3 experiments. ^**^P<0.01 (unexposed control Vs TiO_2_-NPs exposure). ##P<0.01 (TiO_2_-NPs exposure Vs DMTU/NAC treatment). (**b**) The cells were exposed to MWCNTs (10 & 50 µg/ml) with or without DMTU (10 mM) and NAC (2 mM) for 24 h. Ethyl methanesulfonate (6mM) was used as a positive control. Each data point represents the mean of three slides. A total 1000 bi-nucleated (BN) cells with well-defined cytoplasm were scored. Parallel sets were also run under identical conditions by exposing the cells only with either of DMTU or NAC. This group was used to compare the data between scavenger treated Vs non-scavenger treated cells. All values are mean ± S.E. of 3 experiments.^ **^P<0.01 (unexposed control Vs MWCNTs exposure). ##P<0.01 (MWCNTs exposure Vs DMTU/NAC treatment).

Results of MN formation in A549 cells are shown in Figure 5 a & b. Exposure of both TiO_2_ and MWCNTs induces significant number of MN in A549 cells in 24 h of exposure. In case of TiO_2_-NPs, response was dose dependent i.e. (12.66±0.66 & 17.33±0.33) at 10 & 50 µg/ml respectively, whereas the lower dose of MWCNTs (10 µg/ml) induced more MN (16.00±0.57) than higher dose (50 µg/ml) i.e. (13.66±0.33). Both DMTU and NAC were found to be effective in reducing MN significantly (p<0.01). The response of DMTU was greater against TiO_2_-NPs than MWCNTs, whereas, NAC has shown better results against MWCNTs. Though, the effect of both DMTU and NAC were statistically significant (p<0.01) but the values were still greater than the unexposed control cells throughout the exposure concentrations (Figure 5 a & b). The difference in MN induction was insignificant between scavenger treated Vs non-scavenger treated groups.

### Western blot analysis

**Figure 6:**
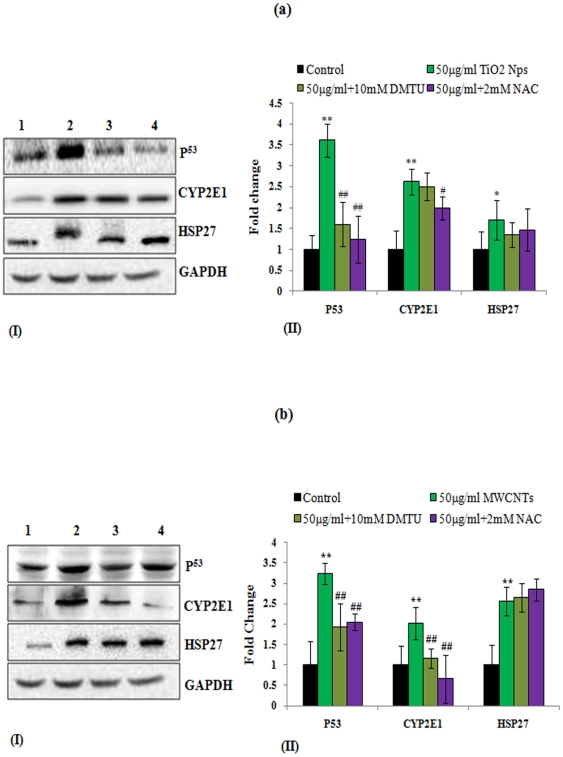
TiO_2_-NPs and MWCNTs (50 µg/ml) induced translational changes (HSP27, CYP2E1 and P^53^) in the absences or presence of DMTU (10 mM)/ NAC (2 mM). **a (I)** Assessment of altered expressions of proteins involved in the induction of oxidative stress (HSP27 and CYP2E1) and genotoxicity (P^53^) in A549 cells exposed to TiO_2_-NPs (50 µg/ml) for 24 h. GAPDH was used as internal control to normalize the data. Lane (1) unexposed control (2) 50.0 µg/ml TiO_2_-NPs (3) 50.0 µg/ml TiO_2_-NPs + DMTU (10 mM) (4) 50.0μg/ml TiO_2_-NPs + NAC (2 mM). **(II)** Relative quantification (fold change) of proteins involved in the induction of oxidative stress (HSP27 and CYP2E1) and genotoxicity (P^53^) in A549 cells exposed to TiO_2_-NPs for 24h. GAPDH was used as internal control to normalize the data. Quantification was done in Gel Documentation System (Alpha Innotech, USA) with the help of AlphaEase^TM^ FC StandAlone V.4.0 software. All values are mean ± S.E. of 3 experiments. ^**^P<0.01 (unexposed control Vs TiO_2_-NPs exposure). ##P<0.01 (TiO_2_-NPs exposure Vs DMTU/NAC treatment). **b (I)** Assessment of altered expressions of proteins involved in the induction of oxidative stress (HSP27 and CYP2E1) and genotoxicity (P^53^) in A549 cells exposed to MWCNTs (50 µg/ml) for 24 h. GAPDH was used as internal control to normalize the data. Lane (1) unexposed control (2) 50.0 µg/ml MWCNTs (3) 50.0 µg/ml MWCNTs + DMTU (10 mM) (4) 50.0μg/ml MWCNTs + NAC (2 mM). **(III)** Relative quantification (fold change) of proteins involved in the induction of oxidative stress (HSP27 and CYP2E1) and genotoxicity (P^53^) in A549 cells exposed to MWCNTs for 24h. GAPDH was used as internal control to normalize the data. Quantification was done in Gel Documentation System (Alpha Innotech, USA) with the help of AlphaEase^TM^ FC StandAlone V.4.0 software. All values are mean ± S.E. of 3 experiments. ^**^P<0.01 (unexposed control Vs MWCNTs exposure). ##P<0.01 (MWCNTs exposure Vs DMTU/NAC treatment).

In general, exposure of both (TiO_2_-NPs & MWCNTs) at 50 µg/ml induce significant up regulation of genotoxicity and oxidative stress marker proteins, viz., P^53^ (3.61±0.40 & 3.24±0.26 fold), CYP2E1 (2.62±0.30 & 2.02±0.39 fold), HSP27 (1.69±0.47 & 2.56±0.34 fold) respectively. Expression levels of P^53^ protein were found to be reduced significantly (p<0.01) following the treatment of both DMTU and NAC. However, the response was more intense in A549 cells receiving the exposure of TiO_2_-NPs. In case of CYP2E1, cells exposed to MWCNTs shown better responsiveness to the DMTU and NAC with more intense effects of NAC. Contrary to this, the effect of DMTU was non-significant in the cells exposed to TiO_2_-NPs. However, both DMTU and NAC could not restore the expression of HSP27 protein significantly in cells exposed to TiO_2_-NPs/MWCNTs (Figure 6 a & b).

### CYP2E1 dependent n- nitrosodimethylamine-demethylase (NDMA-d) activity

**Figure 7:**
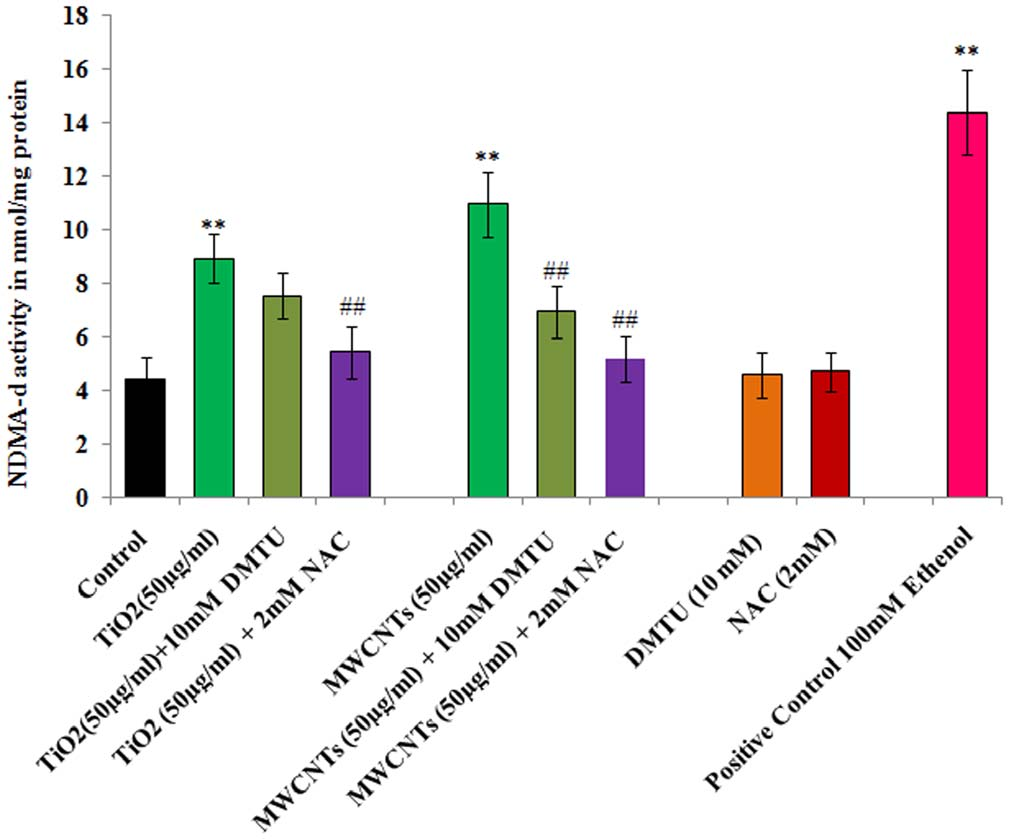
TiO_2_-NPs and MWCNTs (50 µg/ml) induced CYP2E1 dependent n- nitrosodimethylamine-demethylase (NDMA-d) activity in absences or presence of DMTU (10 mM)/ NAC (2 mM). All values are mean ± S.E. of 3 experiments. ^**^P<0.01 (unexposed control Vs TiO_2_ -NPs/ MWCNTs exposure). ##P<0.01 (TiO_2_-NPs/ MWCNTs exposure Vs DMTU/NAC treatment). Specific activity is expressed in nmol HCHO/min/mg protein. Ethanol (100 mM), a known inducer of CYP2E1, was used as positive control.

The data of CYP2E1 dependent catalytic activity are presented in Figure 7. The catalytic activity shows similar trends as observed in western blot analysis for protein expression for CYP2E1 in cells exposed to TiO_2_-NPs and MWCNTs (50 µg/ml). These changes were significant (p<0.001) when compared with unexposed control group as well as to cells treated with DMTU/NAC only i.e., 7.54 + 0.86 and 10.96 + 1.20 nmol /mg protein respectively. Though, both the scavengers show significant restoration in the enzymatic activity in the cells exposed to TiO2-NPs and MWCNTs, but the magnitude o effectiveness was more in case of NAC treatment. Treatment of DMTU or NAC alone did not show any effect on the enzymatic activity when compared with unexposed control group. Ethanol (10 mM), a known inducer of CYP2E1 shows highly significant (p<0.001) increase the NDMA-d activity (Figure 7).

## Discussion

Reactive oxygen species (ROS) mediated oxidative stress has been suggested one among the key events triggers the nanoparticles induced cyto-genotoxicity [25]. We also observed a dose dependent ROS production in A549 cells either exposed to TiO_2_-NPs or MWCNTs at 10 & 50 µg/ml for 24 h. The most important ROS in redox signaling is thought to be H_2_O_2_/OH^•^ radical. Thus, to confirm the ROS redox signaling, cells were exposed to H_2_O_2_ (500 µM) for 1 h under identical conditions, which was served as positive control too. H_2_O_2_ induced similar responses for ROS production as in case of nanoparticles, suggesting implication of similar signal transduction pathways. The reduction of ROS with pre/co-treatment of DMTU and NAC in A549 cells, further confirm the involvement of ROS in the process of toxicity and is in agreement with previous studies [4], [8]. NAC, a precursor of glutathione (GSH), is an important cellular antioxidant and plays a major role in protecting the cells against oxidative stress and inhibits the formation of H_2_O_2_ *in vitro* [23], [26]. NAC treatment causes improvement of cellular GSH level by directing cystine in to the GSH synthesis pathway, because cystine is a common precursor of both NAC and GSH [24]. While the great reactivity of the DMTU molecule toward OH^•^ is due to the extremely favorable electron-donating characteristics of the sulfhydryl group [22].

In the present study, the cytotoxicity of TiO_2_-NPs and MWCNTs was found to be dose and time dependent. In case of TiO_2_-NPs, treatment with DMTU and NAC reduces the cytotoxic effect, suggesting that both the OH^•^ radicals (ROS) generation and reduction in antioxidant defense system are responsible for the decreased viability. While in case of MWCNTs the cytotoxicity was significantly reduced by NAC treatment. It is noteworthy that DMTU, a more specific hydroxyl radical scavenger, was not effective in scavenging hydroxyl radicals in our measurements. This, simplifies that MWCNTs causes toxicity by altering/hampaering the enzymatic antioxidant system and not by directly inducing OH^•^ radicals in the A549 cells. The results suggest that the fraction of ROS that is inhibited by NAC, but not by DMTU, could be a different species of free radicals and also the difference in behavior of both (TiO_2_ and MWCNTs) could be because of their difference in surface structure [27] **,** length [28], and presence of metal catalyst [29] etc. We have also reported earlier that MWCNTs significantly reduced glutathione levels in dose and time dependent manner [15]. It has been postulated that the loss of GSH may compromise cellular antioxidant defenses and lead to induction of ROS [30].

Further, NAC treatment provided sufficiently strong antioxidant defense system, resulting the increased cell viability of cells exposed to TiO_2_ and MWCNTs. Similar kind of effect was observed with human lung epithelial and airway epithelial (HEp-2) cell line, when co-treated with antioxidant NAC and Resverartrol against ZnO and CuO nanoparticles respectively [19], [21].

Both type of nanoparticles (TiO_2_ and MWCNTs) are known to induce Micronuclei (MN) in dose dependent manner, which is well defined biomarker for genotoxicity assay [31]. Our findings of MN induction are well consistent with earlier studies using nano-TiO_2_ in lymphoblastoid cells [32], and in rat lung epithelial cells exposed to MWCNTs [33]. The protective effect of DMTU (10 mM) and NAC (2 mM) on MN induction could be observed clearly in the present investigations (Figure 5 a & b). Our MN studies further strengthen the role of oxidative stress in genotoxicity induced by TiO_2_-NPs and MWCNTs in A549 cells. In the previous investigation crocidoloite and chrysotile asbestos fibers also have been reported to induce MN, which significantly decrease after treatment with NAL, a synthesized salt of N-acetylcysteine (0.2 mM) and DMTU (20 mM) in human mesothelial cells [34].

In the present investigations, western blot analysis was done to assess the altered expression of proteins such as HSP27 and CYP2E1, indicators of oxidative stress, and P^53^ as a biomarker of genotoxicity. Heat shock proteins (HSPs) comprise several different families of proteins that are induced in response to wide variety of physiological and environmental insults. One such protein is HSP27, which is induced highly during stress conditions. HSP27 is known to interfere with ROS induced key components of apoptotic signaling pathway, and is correlated with increased survival in response to cytotoxic stimuli [18]. Our study reveals that at 50 µg/ml concentration, both the nanoparticles (TiO_2_-NPs and MWCNTs) induced significant expression of HSP27, suggesting that the cells were under stress. The treatment of DMTU and NAC did not show significant alterations in the nanoparticles induced expression of HSP27. It indicates that either cells are still under stress conditions or DMTU and NAC reduce the oxidative stress by some other route under our experimental conditions. Further experiments with different range of time points, exposure concentrations, etc. are being carried out to explore the significance of elevated levels of HSP27 in presence of DMTU and NAC in cells exposed to these nanoparticles.

The upregulation in the protein expression of CYP2E1 has been correlated with induced formation of ROS, elevation of lipid peroxidation and cytotoxic damage in alcohol induced hepatotoxcity [35], [36]. We report the TiO_2_-NPs and MWCNTs induced induction in the CYP2E1 specific NDMA-d catalytic activity and significant restoration by two scavenges-DMTU and NAC in human lung cancer cells-A549. To the knowledge, nanoparticles induced protein expressions and activity of CYP2E1 and subsequent induced levels of ROS are being presented for the first time in human lung cancer cells A549. Induced levels of CYP2E1 protein expression and NDMA-d activity in present investigations also support our earlier finding in the same cells that MWCNTs induce ROS generation by mixed function oxidase activity rather than mitochondrial disruption [15]. Following the exposure of TiO_2_-NPs and MWCNTs, induced expression and activity of CYP2E1 were observed, which may be correlated with ROS induction and oxidative stress. As our data of ROS generation measured by DCFH-DA are well corroborative with CYP2E1 induction. The similar kind of correlation between ROS production and CYP2E1 following the exposure of monocrotophos, an organophosphate pesticide, in PC12 cells has also been reported recently [37]. We got a significant reduction in the levels of MWCNTs induced oxidative stress and CYP2E1 (expression and activity) in the presence of DMTU and NAC, which confirms the role of CYP2E1 in MWCNTs induced toxic responses. While in case of TiO_2_-NPs, only NAC was found effective in reducing the expression and activity of CYP2E1. It indicates that TiO_2_-NPs exerted the oxidative stress predominantly by H_2_O_2_ production rather OH radicals. As DMTU is known to scavenge the OH^•^ radicals formed by conversion of O_2_^−^ in the presence of iron by a modified Haber–Weiss reaction [38]. Similar effects were also reported in the presence of diallyl-sulfide (another thiol group containing compound) in ethanol mediated expression of CYP2E1 in polarized hepatic cells WIF-B [39].

In the present investigations, the levels of protein expression of P^53^ were well correlative with the data obtained for micronuclei (MN) induction. P^53^ is one of the important triggering molecular markers for assessing genotoxicity in the event of oxidative stress induced DNA damage [10]. Cellular damage by ROS is positively linked with P^53^ activation, and induced expression of P^53^ protein has been suggested as a tool to detect oxidative stress induced cyto-genotoxicity [10]. Thus, the elevated levels of P^53^ protein is an indication of TiO_2_-NPs and MWCNTs induced oxidative stress mediated genotoxicity. Treatment of DMTU and NAC was found to be effective significantly to bring down the elevated levels of p^53^ protein in TiO_2_ and MWCNTs exposed cells. This effect can be correlated with the strong anti-oxidant and anti-ROS activity of DMTU and NAC observed in the present investigations as well as in other studies [34]. In a similar finding by Mroz et al. [40] has also demonstrated that NAC blocked particles driven p-ser15-p^53^ response in A549 cells.

In conclusion, present investigations reveal that TiO_2_-NPs (10, 50 µg/ml) and MWCNTs (10, 50 µg/ml) exposure for 24 h induces the markers of oxidative stress and cyto-genotoxicity in A549 cells. TiO_2_-NPs induced toxic responses were mediated primarily through H_2_O_2_ generation, and reduction in antioxidant defense system. While, in case of MWCNTs, adverse effects were primarily due to altering/hampering the enzymatic antioxidant system. DMTU and NAC were found to be effective significantly in reducing the levels of oxidative stress and genotoxicity markers studied except HSP27.

## Materials and Methods

All the specified chemicals and reagents were purchased from Sigma-Aldrich Chemical Company Pvt. Ltd. St. Louis, MO, USA, unless otherwise stated. All the chemicals and reagents used were of highest purity available.

### Nanoparticles preparation

TiO_2_-NPs (d<25 nm, specific surface area 200–220 m^2^/g, 99.7% pure trace metal basis, tetragonal in crystallographic system, spherical in shape) without any coating were purchased from Sigma Aldrich, USA, (Cat no. 637254), while the MWCNTs used in this study were generously gifted by Dieter G. Weiss, Professor, Department of Biological Sciences, Institute of Cell Biology and Biosystems Technology, Rostock University, Germany. Sterilization of nanoparticles were carried out by heating at 120°C for 2 h and then suspended in complete DMEM-F12 medium. Stock solutions (1mg/ml) of nanoparticles were sonicated to ensure the uniform suspension before being diluted with culture medium used for exposure of cells [15], [41]. Prior using in the experiments, both the nanoparticles were analyzed for the presence of bacterial endotoxin using Limulus Amebocyte Lysate (LAL) test.

### Characterization of nanoparticles

#### Transmission electron microscopy

The ultra-structural characterization including texture, size and internalization was done by Ludwig Jonas, Professor, at Department of Pathology, Electron Microscopic Center, Medical Faculty, University of Rostock, Germany. In brief, TiO_2_-NPs and MWCNTs were suspended in distilled water and dropped on carbon film coated copper grids for Transmission Electron Microscopy. The diameter of TiO_2_-NPs and length/diameter of MWCNTs were measured using TEM Libra 120 (Carl Zeiss Oberkochen, Germany) equipped with morphometric analysis software (iTEM OSIS Münster Germany). Parallel sets of the cells were exposed to TiO_2_-NPs and MWCNTs for 24 h at 37°C, fixed in glutaraldehyde (4% in PBS) and processed to study the phagocytosis and pinocytosis using transmission electron microscopy (TEM). Images were taken at various magnifications by 2K proscan camera using the software iTEM (OSIS Münster,Germany).

#### Dynamic light scattering

Size distribution and zeta potential of TiO_2_-NPs and MWCNTs, alone as well as in the presence of DMTU and NAC, were determined using dynamic light scattering and phase analysis light scattering (PALS) in a Zetasizer Nano-ZS, Model ZEN3600 equipped with 4.0mW, 633nm laser (Malvern instruments Ltd., UK) as described earlier by us [15].

### Cell culture

Human lung cancer cells - A549 (ATCC No. CCL-185TM) used in the study were originally procured from National Centre for Cell Sciences, Pune, India, and grown in DMEM/F-12 (Hams) supplemented with 10.0% fetal bovine serum (FBS), 0.2% sodium bicarbonate, and antibiotic/antimycotic solution (100x, 1 ml/100 ml of medium, Invitrogen, Life technologies, USA). The cells were maintained in 5% CO_2_-95% atmosphere under high humidity at 37°C. Prior using in the experiments, cells were assessed for cell viability by trypan blue dye exclusion assay as describe earlier by us [41] and batches showing viability more than 95% were only used in the experiments.

### Experimental design and exposure condition

**Figure 8:**
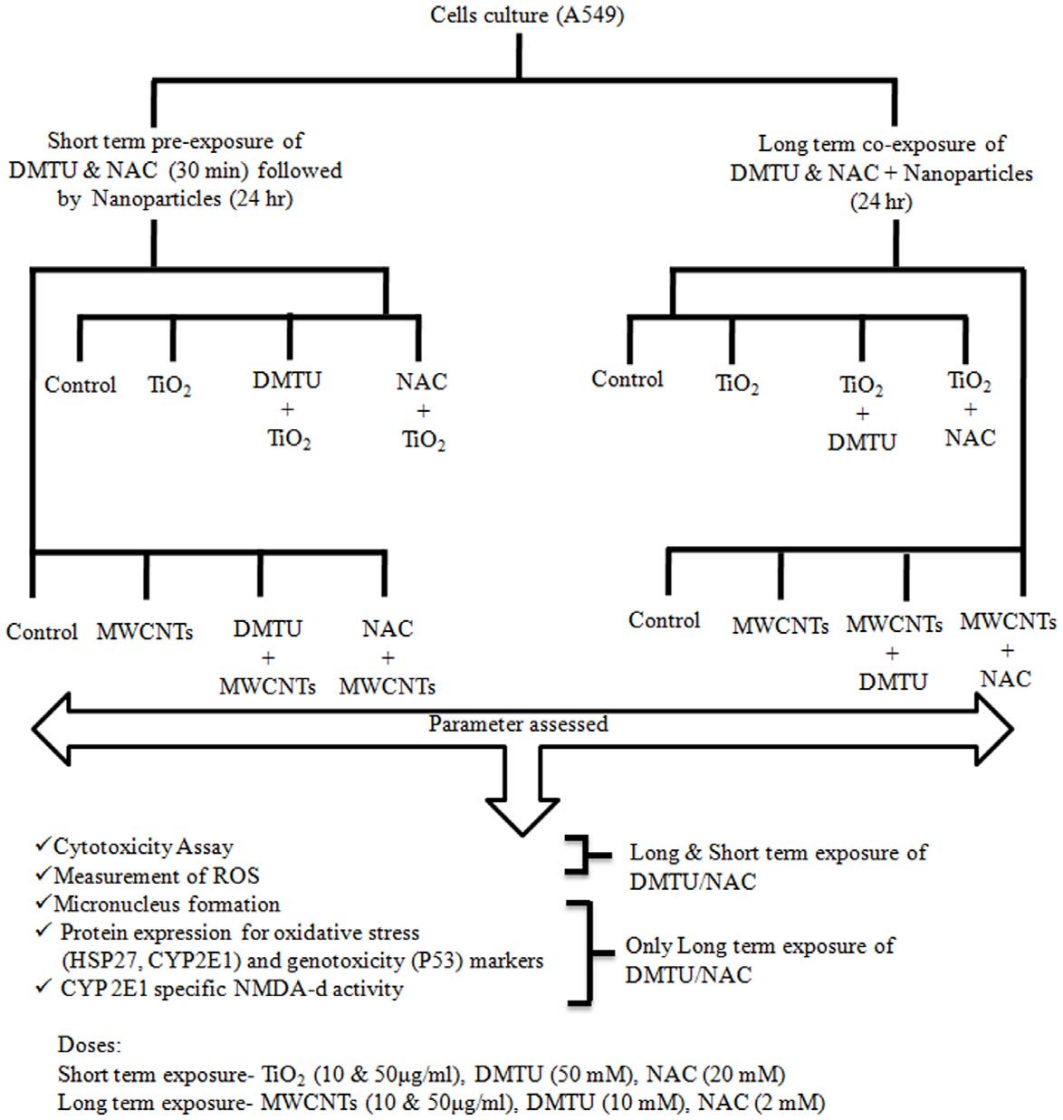
Experimental design. Experimental design showing the exposure protocol of nanoparticles (TiO2-NPs and MWCNTs) and scavenges (DMTU and NAC), study groups and endpoints studied in human lung cancer cells-A549.

Cells were exposed to either of TiO_2_-NPs and MWCNTs (10 and 50 µg/ml) for 24 h in culture medium supplemented with 5% FBS. In short term exposure group, cells received an exposure of either of N-acetlylcysteine (NAC- 20 mM) and 1, 3-dimethyl-2-thiourea (DMTU- 50 mM) for 30 minutes prior to nanoparticles exposure. Whereas, in long term exposure group, cells were treated with DMTU (10 mM) or NAC (2 mM) for 24 h along with nanoparticles exposure (Figure 8). Based on the cytotoxicity data, the doses of nanoparticles and DMTU & NAC were selected in the study (data not shown). The cytotoxicity assessment was done using standard endpoints viz., tetrazolium bromide salt (MTT), neutral red uptake (NRU) and released lactate dehydrogenase (LDH) assays. We describe the MTT assay only, since it was found to be most sensitive among the assays used in the study.

### Restoration of cell viability (MTT assay)

The MTT assay was done as described earlier by us [42]. In brief, cells (1x10^4^ per well) were seeded in 96-well culture plates and allowed to adhere for 24 h in CO_2_ incubator at 37°C. The medium was then aspirated and cells of different groups were subjected to receive the exposure of TiO_2_-NPs or MWCNTs and treatment of DMTU and NAC in low serum (5%) medium as pre discussed in experimental design. Four hour prior the completion of exposure periods (24−4 = 20 h), tetrazolium bromide salt (5 mg/ml of stock in PBS) was added in the plates (10 µl/well in 100 µl of cell suspension) and the plates were then re-incubated for 4 h. The reaction mixture was carefully taken out and 200 µl of DMSO was added to each well and pipetting up and down several times unless the content got homogenized. The plates were kept on rocker shaker for 10 min at room temperature and then read at 550 nm using Multiwell Microplate Reader (Synergy HT, Bio-Tek, USA). Parallel sets without any exposure were also run under identical conditions and served as basal controls.

### Intracellular reactive oxygen species (ROS) generation by DCFH-DA dye

ROS generation was studied by fluorometric analysis following the modified protocol of Kashyap et al. [43]. In brief cells (1x10^4^ per well) were seeded in 96 well black bottom culture plates for 24 h in CO_2_ incubator at 37°C. The medium was then aspirated and cells were exposed to nanoparticles and DMTU or NAC as per stated in the experimental design. On the completion of respective exposure periods, three washings of PBS were given to the cells to ensure the complete removal of extracellular nanoparticles. Then the cells were incubated with 2′, 7′-diclorodihydrofluorescein di-acetate (DCFH-DA, Sigma Aldrich) (10 µM) for 30 min at 37°C. The reaction mixture was then aspirated and cells were washed three times in PBS and finally placed in 200 µl of PBS/well. Fluorescence intensity was measured using multiwall micro plate reader (Synergy HT, Bio-Tek, USA) on excitation wavelength at 485 nm and emission wavelength at 528 nm. Hydrogen peroxide (H_2_O_2_, 500 µM) was used as a positive control 1h prior to completion of exposure time.

### Cytokinesis block micronucleus (CBMN) assay

The micronucleus assay was done as described earlier by us [44]. The cells were exposed to TiO_2_-NPs and MWCNTs (10 & 50 µg/ml) with or without DMTU (10 mM) and NAC (2 mM) for 24 h, then cells were washed and supplemented with cytochalasin B (3 µg/ml, Sigma)-containing medium and were incubated further for another 22 h to accomplished the nuclear division (22h-doubling time for A549 cell line). Further, cells were washed in hypotonic buffer (0.56% KCl) for 10 min and fixed in Carnoy's fixative (methanol: acetic acid, 3∶1). Ethyl methanesulfonate (EMS Cas No. 62-50-0) was used as a positive control. The slides were then prepared, stained with Giemsa, and mounted with DPX for microscopy to examine the presence of micronucleus (MN). Each data point represents the mean of three slides. A total 1000 bi-nucleated (BN) cells with well-defined cytoplasm were scored. Parallel sets were also run under identical conditions by exposing the cells only with either of DMTU or NAC. This group was used to compare the data between scavenger treated Vs non-scavenger treated cells.

### Translational changes in oxidative stress and genotoxic markers

Western immunoblotting was done to assess the TiO_2_-NPs and MWCNTs (50 µg/ml) induced translational changes in the absences or presence of DMTU (10 mM) and NAC (2 mM) for the expression of selected markers associated with oxidative stress viz., HSP27 CYP2E1 and genotoxicity P53. Cells were pelleted and lysed using CelLyticTM M Cell Lysis Reagent (Cat No# C2978, Sigma, USA) in the presence of protein inhibitor cocktail (Cat No# P8340-5ML, Sigma, USA). Protein estimation was done using BCA Protein Assay Kit (Catalogue # G1002, Lamda Biotech, Inc., St. Louise, MO, USA). Then denatured proteins (50 µg/well) were loaded and electrophoresed using 10% Tricine-SDS gel. Proteins were transferred on Immobilon-P membrane (Millipore Cat No. IPVH00010, USA) at 180 mA current for 3 h. Nonspecific binding was blocked with 5% nonfat dry milk powder in TBST [20 mM Tris-HCl (pH 7.4), 137 mM NaCl, and 0.1% Tween 20] for 2 h at 37°C. After blocking the membranes were incubated overnight at 4°C with anti-protein primary antibodies specific for p^53^ (1∶1000, Chemicon, USA), HSP27 (1∶5000, Santa Cruz, USA), CYP2E1 (1∶1000, Chemicone, USA) and GAPDH (1∶1000, Chemicon, USA) in blocking buffer (pH 7.5). The membrane was then incubated for 2 h at room temperature with secondary anti-primary antibody conjugated with horseradish peroxidase (Calbiochem, USA). Then the blots were developed using by luminol (cat no 34080 Thermo Scientific, USA) and densitometry for protein specific bands was done in Gel Documentation System (Alpha Innotech, USA) with the help of AlphaEaseTM FC StandAlone V.4.0 software.

### CYP2E1 dependent n- nitrosodimethylamine-demethylase (NDMA-d) activity

The catalytic activity of cytochrome P450 2E1 was measured by the N-demethylation of N-nitrosodimethylamine (NDMA), known to be specifically catalyzed by CYP2E1in human lung cancer cells-A549 following the protocol described earlier by Kapoor et al. [45]. The assay mixture contained 70 mM Tris–HCl (pH 7.4), 10.0 mM semicarbazide, 14 mM MgCl_2_, 215 mM KCl, 4 mM NADPH, and a suitable amount of protein in final volume of 1.0 ml. The reaction was carried out by incubating the mixture at 37°C for 30 min and stopped by the addition of 1.5 ml of 12.5% trichloroacetic acid (TCA). After centrifugation at 2000 rpm for 1.5 min, 2 ml of supernatant was mixed with 1.0 ml of NASH reagent containing 6 M ammonium acetate, 60 mM acetyl acetone and 0.15 M acetic acid. The tubes were then incubated at 70°C for 20 min and HCHO formed was measured at 415 nm. Protein content of the samples was estimated using BCA Protein Assay Kit (Catalogue # G1002, Lamda Biotech, Inc., St. Louise, MO, USA). The specific catalytic activity is expressed in nmol HCHO/min/mg protein.

### Statistical analysis

Results were expressed as mean ± standard error of mean (SEM) for the values obtained from at least three independent experiments. Statistical analysis was performed using one-way analysis of variance (ANOVA) and post hoc Dunnett test to compare the control vs. treated group by SPSS Version 12. The values p<0.01 was considered as significant.
